# TP-Net: Self-Supervised Pretrained Network Traffic Representations for Few-Shot Threat Detection in IoT and Sensor Networks

**DOI:** 10.3390/s26216861

**Published:** 2026-10-30

**Authors:** Xi Yao, Yan Feng, Qianchu Wang

**Affiliations:** School of Information Science and Technology, Xizang University, Najin Road, Lhasa 850000, China; 13574913636@163.com (X.Y.); qchuchu@163.com (Q.W.)

**Keywords:** IoT security, sensor network traffic classification, self-supervised contrastive learning, few-shot learning, attack detection, encrypted traffic

## Abstract

Encrypted traffic now dominates IoT and sensor networks, and fully supervised classifiers are bottlenecked by label scarcity and the retraining cost of recognizing new attacks at the resource-constrained edge. TP-Net (Traffic Pretraining Network) couples self-supervised contrastive pretraining with *N*-way *K*-shot evaluation: a four-branch 1D-CNN encoder is trained once under SimCLR on packet, burst, flow-statistical, and TLS-handshake features with five traffic-specific augmentations, and it is then frozen at deployment. Our contributions are threefold. First, we build a protocol-fair benchmark that evaluates six supervised-traffic baselines and TP-Net under the same per-episode linear head with a frozen encoder, showing that TP-Net’s SSL encoder stays competitive, trailing the strongest fully trained supervised model by at most 9.75 pp, without any encoder fine-tuning while supervised baselines require retraining. Second, we demonstrate novel-class and encrypted-only generalization, reaching 99.3%/72.9% on unseen novel attacks (CIC two-way/USTC five-way; ISCX’s one-way split is trivial and excluded) and +28.66 pp over no pretrain on fully encrypted DoHBrw-2020 traffic. Third, we show that augmentation strength must adapt to task complexity, overturning the canonical SimCLR stronger-is-better rule on IoT traffic. TP-Net lifts five-shot accuracy over a random encoder by +13.14/+7.76/+4.11 pp and yields a 5-alert, no-finetune, hour-level deployment path for novel encrypted attacks at the edge.

## 1. Introduction

### 1.1. Background and Motivation

Encrypted traffic now accounts for more than 90% of network traffic [1]. Attackers have accordingly migrated command-and-control (C2) communications and malicious payloads to TLS, DoH, QUIC, and other encrypted channels. Against these channels, traditional deep-packet-inspection and plaintext-payload solutions such as Snort [2] and Suricata are largely ineffective. As discussed in the classic network security literature [3], the reliance of these tools on plaintext payload inspection makes them fundamentally unsuited for modern encrypted environments. Even for traffic that remains partially plaintext, increasingly complex application-layer encapsulation keeps the maintenance cost of DPI prohibitively high.

Behavior-based traffic analysis has therefore become the prevailing direction. By inferring the traffic category from packet-length sequences, burst patterns, flow statistics, and handshake features (e.g., JA3), it is well suited to the dominant case of encrypted traffic, which is the most salient driver of this shift. Representative works in this direction include FS-Net [4], YaTC [5], ET-BERT [6], and TFE-GNN [7]; see [8] for a survey. These methods, however, generally struggle with novel attacks: new-attack alerts typically number in the single digits, making full supervision expensive to label. Moreover, the learned features are coupled to the training-set distribution and transfer poorly to unseen attacks; traditional architectures further require full retraining, which cannot keep pace with attackers’ rapid turnover of C2 domains and payloads. The central methodological challenge underlying these limitations is how to endow a traffic-detection model with both few-shotcapability and *strong generalization* (as also addressed in this work).

This challenge is particularly acute in IoT and sensor networks where devices are deployed in resource-constrained edge environments with limited computation and intermittent cloud connectivity. Unlike general-purpose enterprise traffic, IoT sensor traffic shows highly periodic telemetry, limited payload diversity, and strict latency requirements for local anomaly detection [9]. Recent work shows that self-supervised embeddings extend to these settings, where resource constraints and new attack surfaces demand lightweight yet semantically enriched intrusion detection [9]. The retraining-heavy supervised paradigm of conventional traffic classifiers is therefore impractical at the edge, which amplifies the value of a pretrained, frozen encoder that recognizes novel threats from a handful of labeled alerts. TP-Net is designed for this setting: one-time SimCLR pretraining produces a frozen four-branch encoder that runs in real time on commodity gateways, while novel attacks are absorbed by fitting a one-line sklearnLogReg on K=5 support samples without encoder retraining.

### 1.2. Gap with Existing Work

The systematic differences between existing research and the proposed TP-Net (Traffic Pretraining Network) span five dimensions, summarized in Table 1. A core distinction from the “SSL followed by full supervision” paradigm (as in ET-BERT) is that, in TP-Net, the encoder is no longer updated after the pretraining stage, and the downstream evaluation is conducted exclusively through *N*-way *K*-shot episodes, forming a complete SSL–few-shot closed loop rather than using SSL merely to accelerate fully supervised training.

### 1.3. Contributions

This paper makes three principal contributions, matching the structure of the Abstract. The individual building blocks—SimCLR, a ProtoNet-style few-shot head, and a multi-branch 1D-CNN encoder—are established techniques; TP-Net’s novelty therefore lies in the systematic combination and evaluation of these components, not in inventing any single component: (i) the protocol-fair evaluation that isolates what the SSL representation itself delivers, and (ii) the traffic-specific empirical findings below.

**C1: A protocol-fair benchmark under a frozen-encoder evaluation head.** We build a unified evaluation protocol that places six supervised-traffic baselines (ET-BERT, FS-Net, YaTC, 1D-CNN, NetMamba, TFE-GNN) and TP-Net under the same per-episode sklearn LogReg ($C=10^{3}$) head on *K* support samples, with every baseline’s encoder frozen after supervised training on the same 20 k stratified subset. We show that TP-Net’s SSL encoder remains competitive without any encoder fine-tuning, whereas supervised baselines require retraining for new attacks. To rule out the concern that a short budget under-trains the baselines, a full-budget sanity check retrains the two strongest baselines (YaTC, TFE-GNN) for 50 epochs; the following conclusion holds: TP-Net SSL FT-Head places in the competitive-middle band on every dataset, trailing the strongest fully trained supervised model (YaTC, 50 ep) by at most 9.75 pp.
**C2: Novel-class and encrypted-only generalization.** We demonstrate that the framework generalizes to completely unseen novel attack classes: CIC 2-class novel reaches 99.3% five-shot accuracy, and USTC 5-class novel reaches 72.9%. Importantly, the same SSL encoder retains its advantage on a fully encrypted IoT workload. On the DoHBrw-2020 DNS-over-HTTPS subset (a common channel for IoT devices to reach C2 servers), TP-Net SSL two-way five-shot reaches 0.838 vs. the no-pretrain random encoder’s 0.551 (+28.66 pp). This rules out the failure mode in which the SSL gain on the three primary datasets is a side-effect of plaintext payload access.
**C3: Task-adaptive augmentation strength.** We show that the canonical “stronger-is-better” SimCLR augmentation rule *does not transfer to traffic data* unmodified. The optimal preset is task-dependent: none for the 2-class ISCX task, medium for the 10-class CIC task, and weak for the 20-class USTC task. The full 3×4 ablation (3 datasets × 4 augmentation presets) and the cross-dataset CKA/transfer matrix are reported in Section 4.4.

## 2. Related Work

### 2.1. Behavior-Based Traffic Classification, SSL, and FSL Background

Traffic classification has shifted toward behavior-based features because payload parsing is either infeasible or too costly, and encrypted traffic is the most salient driver of this shift. FS-Net [4] trains an encoder–decoder to reconstruct packet-length sequences; YaTC [5] applies a Transformer over 2D traffic matrices; ET-BERT [6] performs MLM pretraining within a BERT framework; TFE-GNN [7] uses a graph neural network over burst topology; and NetMamba [10] introduces a state-space model (Mamba) for long-range dependencies. All of these methods rely on large amounts of labeled data and require retraining for new attacks.

Self-supervised contrastive learning [11,12,13] and few-shot learning [14] provide two complementary levers. Contrastive SSL spans three families: *in-batch negatives* (SimCLR [15], MoCo [16]), *negative-free bootstrapping* (BYOL [17], SimSiam [18]), and *reconstructive generative* (MAE [19]). We adopt SimCLR because in-batch contrast directly models the “local similarity” of traffic data (samples from the same application within a batch naturally form clusters), it requires no momentum queue, and its contrastive loss plateaus after epoch 20, avoiding unnecessarily long training. Few-shot learning follows three routes—metric learning (ProtoNet [20], MatchingNet [21]), meta-learning (MAML [22]), and learnable distance (RelationNet [23]). We adopt ProtoNet as the default evaluation paradigm because it is parameter-free, deterministic, and naturally compatible with the SSL philosophy of “learning representations, not classifiers.”

### 2.2. SSL/FSL for Traffic Analysis and the IoT Positioning of This Work

Several recent works apply SSL or few-shot learning to network traffic classification. ET-BERT [6] and Flow-MAE [24] stop at fully supervised downstream; YaTC, FS-Net, and other representative works all depend on large amounts of labels. Improved paradigms for few-shot encrypted-traffic classification include in-context-learning-based ICT-META [25], contrastive-driven meta-learning [26], a pretraining–contrastive-learning synergy for few-shot novelty detection [27], payload-free open-set prototype rejection (SepSpace) [28], SSCL-IDS [29], and MAML combined with contrastive learning [30]; recent surveys [31,32] characterize this emerging line. Most works focus on a single component (either SSL or FSL alone); a systematic evaluation, under one unified protocol, of the synergy among SSL representations, FSL heads, and traffic-specific augmentations remains limited—a gap filled by this work as well.

In this regard, the closest approach that combines a Deep Infomax SSL encoder with Prototypical Networks and a downstream Random Forest for IoT malware image classification on MaleVis and WSN-DS is reported in [33] by Ben Atitallah et al. Their line of work shares the SSL + ProtoNet + IoT positioning that motivates TP-Net but differs on three deployment-relevant respects. On the evaluation protocol, Ben Atitallah et al. fine-tune ProtoNet on each few-shot episode, whereas TP-Net keeps the encoder frozen and fits only a one-line sklearn LogReg on *K* support samples; this avoids the hour-to-day fine-tuning cost that IoT gateways cannot afford. The SSL objective also differs: Deep Infomax maximizes mutual information between local and global features, while TP-Net uses SimCLR in-batch contrastive learning that is closer to the closed-form few-shot prototype geometry. Their method processes malware visualizations (gray-scale image renderings of executable bytes) rather than the four-modality traffic-feature inputs (packet, burst, flow statistics, TLS handshake) that TP-Net uses, so the deployment scenarios are complementary rather than overlapping.

This direction is of major interest for the IoT and sensor-network community. Liu & Guo [9] recently showed that self-supervised semantic embeddings extend to IoT and WSN intrusion detection, achieving 98.5% accuracy on a 100K-record China Mobile dataset and motivating “lightweight yet semantically enriched” IDS for resource-constrained WSNs. Lu et al. [34] proposed the Contrastive Dual-Task Framework (CDTF), combining supervised-triplet pretraining with self-supervised dynamic-burst masking, evaluated on seven public IoT datasets in MEC environments (+4.61 pp Precision under few-shot). These works share TP-Net’s high-level motivation (frozen-encoder few-shot detection for encrypted IoT traffic) but differ from TP-Net on two protocol-level respects. On the pretraining objective, CDTF uses a hybrid supervised + self-supervised objective that requires base-class labels, while TP-Net uses pure-SSL SimCLR with zero base-class labels. On the evaluation protocol, CDTF is evaluated under standard supervised fine-tuning, while TP-Net is evaluated under a strictly frozen-encoder *N*-way *K*-shot episode protocol that more faithfully reflects the IoT-edge deployment scenario where encoder retraining is impractical. The present work therefore complements the IoT SSL/FSL line by quantifying what a strictly frozen-encoder, pure-SSL pipeline can achieve on IoT-relevant traffic with only K=5 labeled alerts per novel class.

## 3. Materials and Methods

The overall architecture of TP-Net is illustrated in Figure 1. The pipeline proceeds in four stages. The input flow is decomposed into four feature views and fed through a four-branch encoder to extract a 128-dimensional embedding. Two stages then follow: offline SSL pretraining, performed once, and online few-shot evaluation with a frozen encoder (five labels per new attack).

### 3.1. Threat Model and Deployment Assumptions

We consider a single-tenant IoT-edge gateway that sits at the boundary between a (possibly encrypted) local sensor network and the wider internet. Three operational conditions shape the threat model.

With respect to adversary capability, the attacker controls the contents of the traffic seen at the gateway but not the gateway itself. Concretely, the attacker can (i) craft arbitrary payloads and packet sequences within the application-layer protocol; (ii) tunnel malicious traffic over encrypted channels such as TLS, DoH, or QUIC; and (iii) rotate C2 domains and TLS fingerprints at a faster cadence than the operator can collect labeled samples. The attacker does *not* have direct access to the gateway hardware, the pretrained encoder, or the operator’s labeling pipeline.

The defender operates a trusted gateway with limited but adequate computational resources: a commodity IoT-class CPU suffices for one forward pass of the four-branch 1D-CNN (millisecond-level latency per flow) and for fitting a one-line sklearn LogReg on *K* support samples per class. The gateway can collect a small number (we assume K∈{1,2,3,5,10}) of operator-confirmed alerts per novel attack class without retraining the encoder. We assume the SSL pretraining corpus is assembled offline from sources the operator trusts (the captured background traffic of the same deployment plus any relevant public datasets) and is not adversarially poisoned at the point of pretraining; an extension to certified-robust SSL pretraining is left to future work.

We do not consider adversarial packet-length perturbations, feature obfuscation that breaks our four-modality pipeline, or firmware compromise of the gateway. Robustness to such conditions requires dedicated defenses (e.g., adversarial training, encrypted-traffic anomaly detection) that are orthogonal to the SSL + few-shot design studied here.

### 3.2. Problem Definition and Architecture

We represent a single encrypted network flow using four feature modalities: $P\in R^{2\times 200}$ (packet sequence, each packet binarized as [direction, length]), $B\in R^{16\times 4}$ (burst [size, time]), $S\in R^{32}$ (flow statistics, IAT, byte/pkt count), and $H\in R^{71}$ (handshake: 6-d JA3 + 32-d cipher suites + 32-d extensions + 1-d miss flag). The objective is to learn an encoder $f_{\theta }\left(\cdot \right)$ such that flows of the same class are close in the embedding space and only *K* labeled samples are required to classify a novel attack. The task is traffic *classification*—assigning each flow to an application or attack class—rather than binary malicious-vs.-benign detection; TP-Net’s security value lies in recognizing newly appeared classes from few labeled flows, not in claiming that every classified flow is itself malicious.

Figure 2 details the four-branch encoder: each branch processes one modality independently, and a fusion MLP concatenates and integrates the four branch outputs into a 128-D embedding.

### 3.3. Augmentation and SSL Pretraining

Figure 3 shows the five traffic-specific augmentations proposed in this work. Each augmentation simulates a real-world network perturbation. The none preset zeroes all augmentation probabilities; although the positive pair then degenerates to an identity mapping, the 2(B−1) negative pairs in the batch and the random subsampling performed at data-loading time still provide a discriminative signal. The empirical benefit of none on ISCX is reported in Section 4.4.

The contrastive loss is the standard NT-Xent (Equation (1), τ=0.1); for each sample *x* in the batch, two augmentations $x_{i}$, $x_{j}$ form the positive pair and all other in-batch samples serve as negatives. The 128-D encoder output is projected into the contrastive space through a 2-layer MLP (128→128→128) used only during SSL and discarded at evaluation time. Optimization uses AdamW with $\mathrm{lr}=3\times 10^{-4}$, weight decay $1\times 10^{-4}$, cosine schedule, batch size 256; MoCo additionally maintains a 4096-entry momentum queue. Training stops after 30–50 epochs because the NT-Xent loss plateaus beyond epoch 20. Moderate temperature τ=0.1 suffices because traffic data has small intra-class and large inter-class variance.

(1) $$L_{\text{NT-Xent}}=-\log\frac{\exp(\mathrm{sim}(z_{i},z_{j})/\tau )}{\sum_{k\neq i}\exp(\mathrm{sim}\left(z_{i},z_{k})/\tau \right)}$$

### 3.4. Few-Shot and Novel-Class Evaluation Protocols

In deployment, novel attacks have only a handful of labeled samples (5–50); the conventional train/val/test split cannot faithfully reflect this “small-sample” scenario. The *N*-way *K*-shot episode protocol is therefore closer to operational needs. At each round, *N* classes are sampled uniformly at random; for each, *K* support and *Q* query samples are drawn, the encoder computes a prototype from the support set, and accuracy is measured on the queries (Figure 4, Equation (2)). We adopt the cosine-distance variant of ProtoNet (d=1−cos) for stability at small *K*; MatchingNet is also evaluated as a comparator (see Section 4.2).

(2) $$c_{k}=\frac{1}{K}\sum_{i=1}^{K}f_{\theta }(x_{i}),\;p\left(y=k|x\right)=\frac{\exp(\cos\left(f_{\theta }(x),c_{k})/\tau \right)}{\sum_{k^{\prime }}\exp(\cos\left(f_{\theta }(x),c_{k^{\prime }})/\tau \right)}$$

where $c_{k}$ is the mean prototype of class *k* over the support set and $f_{\theta }\left(\cdot \right)$ is the frozen encoder.

Real deployment also requires that novel attacks never appear in the pretraining data. We therefore partition classes by taking the last *N* classes in sorted label order as novel, leaving the rest as base: only base-class samples participate in SSL pretraining (label-free), and during evaluation novel classes are mixed into the support/query sets to verify whether they can be recognized from only K=5 labels despite never having been seen. This yields 8/2 (CIC, novel: PortScan, SSH-Patator), 15/5 (USTC, novel: Tinba, Virut, Weibo, WorldOfWarcraft, Zeus), and 1/1 (ISCX, novel: NonVPN) base/novel splits; a second, independent novel-class partition (3-novel-CIC and 6-novel-USTC) is used for the supplementary SSL-vs.-supervised decision test in Section 4.5. Terminologically, “novel-class few-shot” follows the generic FSL nomenclature [14] (novel classes appear only at evaluation but *K* supports are still provided) and is distinct from strict zero-shot learning [35] (no supports at all).

## 4. Experiments

### 4.1. Experimental Setup

We evaluate TP-Net on three public threat datasets (Table 2): CIC-IDS2017 [36] contains 10 classes (mostly plaintext HTTP, with HTTPS subsets in some classes); USTC-TFC2016 [37] provides 20 classes of mixed real malware and benign application traffic (including semi-encrypted applications such as BitTorrent and Skype); ISCX-VPN2016 [38] is a VPN binary-classification dataset (all traffic goes through VPN tunnels).

CIC-IDS2017 is dominated by plaintext HTTP classes (e.g., BENIGN, WebAttack, WebDDoS); we keep the original mixed form, consistent with YaTC, ET-BERT, and other baselines. Several CIC-IDS2017 attack classes (Bot, PortScan, DDoS, Infiltration) directly correspond to IoT-device-compromise behavior patterns observed in Mirai-class botnets and IoT scanner activity, making the dataset relevant to IoT-edge threat detection even though it is not exclusively an IoT capture. USTC-TFC2016 mixes real IoT malware captures (Tinba, Nsis-ay, Geodo, Miuref, Htbot) with legitimate application flows (BitTorrent, Skype, Gmail, Facetime). These malware families share behavioral similarities with large-scale IoT botnets such as Mirai [39], and they require robust dynamic analysis techniques [40] rather than static signature matching. USTC-TFC2016 is widely used as an IoT-malware-versus-IoT-application benchmark in MEC-style evaluations [34]. ISCX-VPN2016 captures VPN-tunnel traffic, which is the dominant egress mode for IoT gateways accessing enterprise intranets, and the binary VPN-vs.-non-VPN task closely matches the binary anomaly versus-benign decision that an IoT-edge IDS must make [9]. Encrypted-only validation on DoHBrw-2020 is provided below (DoHBrw-2020 SSL 0.838 vs. Random 0.551, +28.66 pp); DoH is one of the preferred channels through which IoT devices reach C2 infrastructure, so this benchmark is directly relevant to encrypted IoT-threat detection.

All SSL pretraining uses AdamW ($\mathrm{lr}=3\times 10^{-4}$, weight_decay $=1\times 10^{-4}$) with a cosine LR schedule, for 30/50 epochs (CIC/USTC use 50 epochs, ISCX uses 30 epochs owing to its smaller data volume); batch size 256, SimCLR temperature 0.1, MoCo queue 4096; the augmentation default is the medium preset (see Section 3.3). Few-shot evaluation uniformly adopts the 5seeds×600episodes *N*-way *K*-shot protocol, with confidence intervals computed at the seed level. ProtoNet with cosine prototype follows Section 3.4; the FT-Head paradigm uses sklearn LogisticRegression ($C=10^{3}$, lbfgs solver); details are given in Section 4.5. All SSL pretraining and supervised-baseline training were performed on a single Intel Arc 130T GPU (16 GB); SimCLR pretraining on 30k flows takes 23–34 min per dataset, and all baselines use the same hardware and the same 20k stratified subset. Throughout the paper, “encoder-retraining cost” denotes the burden a supervised classifier must pay to adapt to a new attack class—the GPU hours to retrain the encoder plus the labeled samples required—which TP-Net avoids by keeping its encoder frozen and fitting only a one-line LogReg on *K* support samples. Table 3 summarizes the protocol correspondence across every model family used in the paper, making explicit which comparisons share a matched training budget, encoder status, and evaluation head.

### 4.2. Main Experiment: Five-Way Five-Shot Classification

The central question addressed in this section is: when only 5 labeled samples are available for a new attack, how much gain does SSL pretraining bring over random initialization? We evaluate ProtoNet under the five-way five-shot protocol on the three datasets, supplemented by MoCo (to validate the contrastive-loss family), MatchingNet (to validate the distance metric), and LinearProbe (to validate representation quality). The “random baseline” used as a comparison is an encoder with the same architecture but without any pretraining; this setup ensures that the improvement due to SSL is entirely attributable to the pretraining process rather than the architecture. All SSL algorithms use the same 50-epoch (medium preset) configuration (30 epochs for ISCX). Table 4 gives the precise numbers together with the SimCLR-vs.-Random gain column ($\Delta_{\text{Proto-Rand}}$); per-*K* scanning results are reported in Table A1.

Interpretation of Table 5. Under the fair FT-Head protocol, YaTC’s MAE + Transformer pretraining outperforms TP-Net SSL FT-Head on all three datasets. 1D-CNN, NetMamba, and TFE-GNN are within a few percentage points of TP-Net, with TFE-GNN beating it on ISCX (+3.60 pp) but falling below on CIC (−8.76 pp). ET-BERT reaches above-random on CIC but is at chance on USTC and ISCX, indicating that MLM-pretrained bidirectional Transformers do not separate application-class geometry for few-shot classification. FS-Net is essentially at chance on USTC and well below the random encoder on CIC, consistent with its reconstruction objective producing length-statistics-dominated embeddings. Notably, the supervised advantage at the FT-Head does not translate to the frozen-encoder ProtoNet head (e.g., YaTC + ProtoNet on CIC only reaches ∼0.13), confirming that the optimal head is method-dependent. The fact that all supervised-traffic baselines fall below the random-encoder baseline on at least one dataset shows that supervised full-shot accuracy and few-shot transferability are not the same thing.

We further conduct a full-budget sanity check to address the natural concern that 3 supervised epochs under-trains the baselines: we retrained the two strongest supervised baselines (YaTC, TFE-GNN) for a full 50 epochs on the same 20 k subset across all three datasets, with the encoder frozen, then evaluated under the identical FT-Head protocol (3 seeds × 600 episodes). Results are in the bottom block of Table 5. The pattern is dataset-dependent and informative.

On **CIC** (10 classes), 50-epoch training pushes both baselines to near-ceiling accuracy: YaTC 0.9482→0.9897 (+4.15 pp over 3 ep) and TFE-GNN 0.8337→0.9770 (+14.33 pp). At 50 epochs, YaTC sits 6.84 pp *above* TP-Net SSL FT-Head (0.9213) while TFE-GNN overtakes TP-Net by 5.57 pp; the spread is therefore 5.6–6.8 pp on the 50-epoch budget. This serves as a useful upper bound on what supervised pretraining can achieve on the 10-class CIC task.

On **USTC** (20 classes), 50 epochs do not flip the ranking. YaTC 0.8397→0.8443 (+0.46 pp over 3 ep, +3.70 pp over TP-Net); TFE-GNN 0.8106→0.7994 (−1.12 pp), with mild over-fitting visible in the cross-entropy loss rising from 0.50 at ep 30 to 0.55–0.62 at ep 50. TP-Net keeps its USTC lead over TFE-GNN (+0.79 pp) and only loses to YaTC by 3.70 pp.

On **ISCX** (two classes), 50 epochs make YaTC stronger (0.8391→0.8855, +4.64 pp, +9.75 pp above TP-Net) but make TFE-GNN *worse* (0.8240→0.7660, −5.80 pp, −2.20 pp below TP-Net). Over-fitting on a binary task is unsurprising, and TFE-GNN’s pretraining is intrinsically more sensitive to the 2-class geometry.

Taken together, full-budget supervised training *stabilizes YaTC at the top of every dataset and exposes TFE-GNN’s over-fitting risk*, but does not move TP-Net from the competitive-middle band: TP-Net SSL FT-Head requires zero encoder fine-tuning and trails the strongest fully trained supervised model by 6.84 pp on CIC, 3.70 pp on USTC and 9.75 pp on ISCX. The 3-epoch column in the main Table 5 body should therefore be read as a *resource-fair* snapshot. A baseline that underperforms TP-Net in the 3-epoch snapshot does not close the gap with more training, whereas a baseline that beats TP-Net in the snapshot widens its lead at 50 epochs (especially on CIC).

SSL improves consistently on all three datasets, but the gain size tracks task structure: CIC benefits most (+13.14 pp), followed by USTC (+7.76 pp) and ISCX (+4.11 pp), all significant at p<0.001 with pooled Cohen’s *d* of 1.386/0.627/0.338. The non-monotonic pattern reflects what SSL needs to succeed: ISCX’s binary task needs only one decision boundary that a random encoder already approximates (0.651), CIC’s 10 attack classes force the encoder to capture genuine semantics, and USTC’s 20 heavily mixed application classes—whose same-family implementations share packet-length and IAT patterns—blur class boundaries and dilute the benefit. Per-class diagnostics in Section 4.4 confirm this: the gain concentrates on 12–15 malware/mixed-protocol classes, peaking at +53.48 pp for Tinba. The deployment sweet spot is therefore medium-grained multi-class tasks (around 10 classes), where intra-class variance is large and inter-class differences are subtle.

Given that the SSL gain is task-dependent, the next question is which SSL objective and which head to use. SimCLR outperforms MoCo by +1.35 pp on CIC (0.8885 vs. 0.8750), which we attribute to MoCo’s 4096-negative queue introducing false negatives among adjacent flows of the same application. ProtoNet outperforms MatchingNet on all three datasets (+1.25/+7.15/+8.24 pp); our MatchingNet drops the BiLSTM contextualization to fit GPU memory, so it cannot exploit relations among support samples. FT-Head ($C=10^{3}$ LogReg) beats ProtoNet by +9.59/+11.78/+5.06 pp on ISCX/USTC/CIC, mirroring the Δ values in Section 4.5: the gap is largest on USTC (20-class mixing blurs cosine geometry) and ISCX (per-class bias re-calibration helps the binary task). The head should therefore be chosen per dataset: ProtoNet for 5–10 well-separated classes, FT-Head for heavily mixed or binary tasks. The SSL advantage is most pronounced at K∈{1,2,3} (4.6–12.6 pp), which we recommend as the default; at K≥10 the advantage narrows and lighter supervised learning may suffice. The full 4×3 matrix is in Table A1.

Turning to the SSL algorithm and labeling efficiency, the SSL family spans in-batch negative contrast (SimCLR, MoCo), negative-free bootstrapping (BYOL, SimSiam), and reconstructive self-supervision (Embedding-MAE†). The full 3×5 ranking in Table A2 shows SimCLR ranked first on all three datasets, the negative-pair contrastive family ahead of negative-free (+3.8 pp three-dataset average), and Embedding-MAE worst. SSL ProtoNet with K=5 reaches 0.8789, on par with SSL LinearProbe at K=500 (0.8754)—a 100× difference in the number of labels. LinearProbe only becomes advantageous at K≥500; for the zero-day window of a new attack (K∈{1,2,3,4,5}) ProtoNet + SSL encoder is the engineering-optimal solution.

### 4.3. Novel-Class Generalization and Encrypted-Only Validation

We now turn to the novel-class few-shot setting. In Section 4.2, the support and query sets come from the same dataset whose classes all appeared during training. A more realistic deployment scenario is one in which the new attack is completely absent from the pretraining database: when the system first encounters it, only ∼5 alerts are available for labeling. To simulate this, we split each dataset into base/novel parts by taking the last *N* classes in sorted label order as novel; this yields 8/2 (CIC), 15/5 (USTC), 1/1 (ISCX) splits. Novel classes are completely excluded from pretraining (including the augmented views) and mixed into the five-shot support set at evaluation time. CIC’s two novel classes are evaluated as two-way five-shot, USTC’s five novel classes are sampled as five-way five-shot, and ISCX’s one novel class is the trivial one-way five-shot (always 1.0000).

The results (Figure 5): CIC-IDS2017’s last two classes (PortScan + SSH-Patator) reach 0.9931±0.001 under two-way five-shot; USTC-TFC2016’s last five novel classes (Tinba/Virut/Weibo/WorldOfWarcraft/Zeus) sampled as five-way five-shot reach 0.7294±0.003; ISCX-VPN2016’s last one class (NonVPN) reaches 1.0000 under one-way five-shot (trivial). Because ISCX is a binary dataset, its novel split is necessarily one-way and its 1.0000 accuracy is not meaningful evidence of novel-class discrimination; the informative novel-class results are CIC (two-way) and USTC (five-way). On the novel subsets SSL five-shot exceeds the all-class five-shot result on every dataset (CIC 0.993 vs. 0.888; USTC 0.729 vs. 0.692)—the gap reflects reduced task complexity (2–5 classes instead of 10–20), smaller intra-class variance, and the fact that the SSL encoder has already captured the universal traffic fingerprint, so that novel attacks match an existing fingerprint. In real SOC deployments this means that novel attack alerts do not require immediate full retraining: five labeled samples suffice to classify accurately with the SSL encoder. The precise SSL-versus-supervised boundary is dataset-dependent (Section 4.5), and the ability to “learn discriminative representations without task labels” remains a valuable engineering property.

The three primary datasets are dominated by plaintext HTTP, mixed-protocol P2P, or VPN-wrapped traffic, so they cannot by themselves demonstrate that SSL pretraining remains beneficial on a fully encrypted workload. To address this, we additionally evaluate on DoHBrw-2020 [42], which contains only DNS-over-HTTPS (DoH) flows; the encrypted-only benchmark is constructed by collapsing all benign DoH traffic into a single class and treating the malicious_doh subset as a second class, giving a N=2 binary task with ∼6000 flows per class. We pretrain a four-branch SimCLR encoder for 30 epochs on the 12k DoH training split using the medium augmentation preset, freeze it, and evaluate under the two-way five-shot protocol with 5 seeds × 600 episodes. Results are in Table 6.

Even on a fully encrypted DoH workload where the encoder has access only to packet direction, length, IAT, and the (DoH-specific) handshake fingerprint, SSL pretraining still lifts five-shot accuracy by +28.66 pp over a randomly initialized encoder (0.838 vs. 0.551), and the gap is preserved at K=10 (sanity row). We do not interpret this single experiment as evidence that SSL universally dominates on encrypted traffic—DoHBrw-2020 contains only 12k training flows and a binary task, both of which inflate the relative gap—but it does rule out the possibility that the SSL gain on CIC/USTC/ISCX is purely a side-effect of plaintext-payload access. Combined with the TLS-handshake fingerprint branch of the four-branch encoder, the encrypted-only result provides preliminary support that SSL continues to extract useful structure when plaintext inspection is impossible.

### 4.4. Cross-Dataset Transfer and Augmentation Ablation

The “domain-independence” of SSL is one of its central selling points over full supervision: pretraining cost can be amortized across multiple downstream scenarios. To verify this, we pretrain one SimCLR encoder on each source dataset for 30 epochs over 20k samples (stratified), load the resulting encoder directly into the target dataset’s ProtoNet evaluator without any fine-tuning, and evaluate under the strict “zero-adaptation” protocol. The six pairs cover all bidirectional combinations of the three datasets (Figure 6).

The six pairs are full 5-seed × 600-episode measurements: USTC → CIC 0.8284±0.004 (self-training 0.8885, −6.01 pp), CIC → USTC 0.6841±0.003 (self-training 0.6918, −0.77 pp), ISCX → CIC 0.8753±0.004 (−1.32 pp), CIC → ISCX 0.6939±0.006 (+0.18 pp), ISCX → USTC 0.6235±0.005 (−6.83 pp), USTC → ISCX 0.7625±0.007 (+7.04 pp, the only transfer that exceeds self-training). Transfer is therefore *asymmetric*. USTC → CIC (0.8284) outperforms CIC → USTC (0.6841) by 14.4 pp, consistent with the observation that USTC’s 20-class SSL learns more general-purpose “burst pattern + handshake structure” features that remain useful on CIC, while CIC’s attack-oriented features transfer less well. CKA is saturated above 0.99 for all three pairs (USTC/ISCX =0.993, CIC/ISCX =0.996, USTC/CIC =0.989). Pure embedding-space similarity therefore cannot predict downstream transfer accuracy, which spans 25 pp from 0.624 to 0.875. The downstream determinant is the compatibility between source-learned semantics and the target task. Per-class diagnostics further concentrate the USTC gain on 12–15 malware/mixed-protocol classes (Tinba +53.48 pp, Nsis-ay +25.94 pp, Miuref +18.89 pp, Neris +12.98 pp) where SSL captures the “temporal fingerprint” of regular C2 heartbeats, while SSL and Random are nearly indistinguishable on single-protocol application classes (Outlook, WorldOfWarcraft, Skype, Facetime, Δ=−0.14 to −1.77 pp). The full per-class table is reported in Table A4.

Does the SSL consensus of “stronger augmentation is better” still hold in the traffic scenario? For each of the three datasets, we pretrain with the four intensity presets none/weak/medium/strong (see Section 3.3) independently for 30 epochs × 5 seeds × 600 episodes and evaluate with ProtoNet five-shot. All 12 configurations (3 datasets × 4 presets) run a full evaluation independently (Table 7).

The BEST column is counter-intuitive: ISCX (2 classes) chooses none, CIC (10 classes) medium, and USTC (20 classes) weak, so augmentation strength is not a monotonic parameter but a “tuning knob” coupled to task difficulty. none suits ISCX (0.695) because its binary decision rests on a single TLS-handshake fingerprint that strong augmentation can damage; medium suits CIC (0.883) because its 10 attack classes require learning within-class commonality; and weak suits USTC (0.678) because strong augmentation blurs its already-subtle inter-class differences. The effective range is also dataset-dependent—CIC rises monotonically to a plateau (the classic “stronger is better”), USTC is an inverted U, ISCX is reversed—and the practical gain is large on multi-class tasks (CIC/USTC) but limited on ISCX. A practical workflow: train a five-shot baseline with none (∼30 min) as an anchor; if it nears random performance (a hard task with large *N*), switch to weak or medium per Table 7; validate on 1–2 episodes before adopting.

### 4.5. Visualization, Supervised Comparison, and Comprehensive Summary

We provide an intuitive verification via t-SNE projections (Figure A1): for each dataset we sample 1000 flows (stratified), extract 128-D embeddings with the corresponding encoder, and project them to 2D using sklearn t-SNE (perplexity = 30). Under the Random encoder, classes overlap heavily; under the SSL encoder, CIC attack classes form clear clusters (PortScan/DDoS/DoS Hulk/Bot/FTP-Patator), ISCX forms two clean regions (VPN vs. non-VPN), and USTC still exhibits substantial overlap due to the 20-class mixing. Good visual clustering does not equate to high ProtoNet accuracy (e.g., USTC Gmail: SSL 0.512 vs. Random 0.516; per-class diagnostics in Section 4.4). SimCLR pretraining on 30k samples takes only 23–34 min per dataset, well below full-supervision training; this efficiency is the prerequisite for the SOC deployment feasibility discussed in Section 5.2; the corresponding pretraining cost–benefit scatter is given in Figure A2.

The comparison in Section 4.2 is between SSL and Random initialization (same architecture, same parameter count). This paragraph extends the analysis to address two questions: how does SSL compare with supervised pretraining (the same four-branch 1D-CNN trained end-to-end for five epochs with cross-entropy), and under the more deployment-realistic “5-shot fine-tune head” paradigm, does the SSL encoder still outperform Random? Results are in Table 8.

On known classes, supervised outperforms SSL on five-shot ProtoNet for all three datasets (+0.99 to +19.58 pp), with the gap *negatively correlated* with class count—ISCX (2) +19.58 pp, CIC (10) +6.46 pp, USTC (20) +0.99 pp—because five epochs already saturate low-class heads while under-training the 20-class USTC head (0.2676). In the novel-class scenario, SSL is +3.45 pp higher on CIC and supervised +3.99 pp higher on USTC. FT-Head outperforms ProtoNet on all three datasets, so the 128-D SSL representation is highly amenable to a linear classifier and does not depend on ProtoNet’s cosine-prototype geometry. FT-Head is therefore the optimal SOC default: it works at K=5, is a one-line sklearn fit, and supports incremental updates.

The three axes analyzed above—known-class few-shot, novel-class generalization, and cross-dataset transfer—summarize TP-Net’s empirical value, and the corresponding numbers are collected in the public repository.

## 5. Discussion

### 5.1. SSL Effectiveness and Adaptive Augmentation

The framework targets the zero-day window when only ∼5 alerts of a new attack are available at the IoT edge. In this window (Table 8) SSL ProtoNet reaches 0.993/0.729/1.000 on novel classes that were completely absent from pretraining. The supervised-pretraining numbers in Table 5 therefore bound the upper limit of full-shot supervised learning under the same per-episode head, not the comparison target for TP-Net’s primary deployment scenario. Within the SSL-vs.-Random comparison that is the canonical SSL evaluation (Table 4), TP-Net delivers consistent gains; the discussion below explains where and why.

Each network flow exhibits stable structure at four scales: packet-level sequence distribution, burst pattern, flow-level statistics, and TLS-handshake fingerprint. SimCLR constructs two views of the same flow so that the encoder learns what constitutes the same flow; this invariance naturally corresponds to the signature of an application or an attack. Consequently, even when a novel attack arrives without labels, the encoder can relate its fingerprint structure to existing categories. The SSL gain is an inverted-U function of task complexity: it is smallest on the binary ISCX task, largest on the medium-grained 10-class CIC task, and intermediate on the 20-class USTC task, making CIC-like settings the sweet spot for SSL deployment. In the novel-class setting, SSL maintains high accuracy on unseen attacks, so a new attack can often go online with only 5–50 alerts, reducing latency from days to hours.

This behavior differs from that of SimCLR and MoCo on ImageNet. On ImageNet, strong augmentation is essential because intra-class visual variation is large. On traffic data, by contrast, discriminative signals are concentrated in high-information dimensions such as packet length, IAT, and TLS-handshake fingerprints, where overly strong perturbations can destroy critical features. Augmentation strength should therefore be adapted to the number of classes: a 2-class task is best left at none, a 5–10-class task benefits from medium, and a 15+ class task prefers weak. The cross-dataset results further show that transfer is not symmetric: a source encoder trained on a harder task can transfer better to an easier target, while embedding-space similarity alone (e.g., CKA >0.99) is not sufficient to predict transferability.

### 5.2. IoT-Edge Deployment and Limitations

In real IoT-edge deployment the model faces three operational threats detailed in Section 3.1: SSL pretraining poisoning, inference-time adversarial perturbations, and distribution shift from new C2 tool versions. Distribution shift is the most operationally relevant threat: when a C2 tool changes version and its fingerprint no longer matches, TP-Net’s few-shot novel-class adaptation plus continuous micro-fine-tuning can absorb the drift, as five new alerts suffice to rebuild the prototype without retraining the encoder. The resulting security gains are meaningful in principle but have not been benchmarked on a live gateway: the retraining window is estimated to fall from 24–48 h to hour-level five-shot labeling, manual annotation is estimated to fall from thousands of samples to five, and hand-crafted payload signatures no longer need to be maintained. In the open-set setting, the SSL embedding achieves AUROC 0.9127 on CIC and 0.8022 on USTC. Known-unknown is easier to detect than true-unknown. For USTC the large number of classes makes the embedding nearly spherical (confidence gap only 0.07), so Mahalanobis or Energy scoring is needed as an additional signal.

The frozen-encoder architecture offers three engineering properties that are relevant to—though not by themselves sufficient for—IoT-edge deployment, echoing the requirements in the recent IoT-IDS literature [9]. First, the 128-D embedding is a single forward pass of the four-branch 1D-CNN: the encoder has 313 k parameters (1.22 MB in fp32) and 11.32 MFLOPs per flow, with a measured CPU inference latency of 1.70 ms per flow (batch size 1, Intel Core Ultra 5 225H), while pretraining on 30k flows takes 23–34 min per dataset. Second, the fine-tune head is a one-line sklearn LogisticRegression fitted on five support samples per class without GPU acceleration, avoiding GPU-equipped retraining at the edge. Third, a completely frozen encoder allows model updates to be delivered as a single checkpoint download rather than a full retraining pipeline, which matters for devices with intermittent connectivity, limited storage, and over-the-air update constraints.

Compared with CDTF [34], TP-Net’s strict frozen-encoder protocol removes the requirement for any base-class labels during pretraining, which further simplifies the data-collection pipeline at the IoT edge. The encoder can be pretrained on raw sensor traffic dumps with no manual labeling step before being deployed.

Several caveats qualify the SOTA positioning above. The fair SOTA comparison in Table 5 puts YaTC at 0.8855 on ISCX (+9.75 pp over TP-Net); this is the upper bound under the same per-episode head, not the comparison target for the ∼5-alert scenario of Table 8. DoHBrw-2020 remains the only fully encrypted benchmark (N=2), so broader pure-encrypted protocols (QUIC, ECH) and multi-class encrypted workloads await validation. Real IoT-edge deployment still requires gateway ingestion (Zeek/Suricata), ONNX/TensorRT compression of the 128-D encoder, edge alerting, and SIEM feedback loops, all outside this empirical scope. Finally, the four-branch 1D-CNN backbone and the five augmentations are empirical choices whose systematic ablation is left to future work. All validation draws on public lab-captured traces, and the gap between such traces and live IoT-edge deployments has not been quantified.

## 6. Conclusions

This study demonstrates that a frozen self-supervised encoder, pretrained once under SimCLR and evaluated via *N*-way *K*-shot episodes, can recognize unseen encrypted threats at the IoT edge from only K=5 labeled alerts without any encoder retraining. First, the SSL encoder lifts five-shot accuracy over a random encoder by +13.14/+7.76/+4.11 pp on CIC-IDS2017/USTC-TFC2016/ISCX-VPN2016, and under a unified per-episode linear head it remains competitive with fully trained supervised baselines, trailing the strongest (YaTC, 50 ep) by at most 9.75 pp without fine-tuning. Second, the same encoder generalizes to novel-class attacks (99.3%/72.9%; ISCX’s one-way split is trivial and omitted) and to fully encrypted traffic, gaining +28.66 pp over no pretrain on DoHBrw-2020, which rules out plaintext-payload leakage. Third, augmentation strength must be tuned to task complexity rather than assumed “stronger is better”: the optimal preset is none/medium/weak for the 2-/10-/20-class tasks. We stress that TP-Net does not surpass fully supervised models under matched budgets—the strongest baseline (YaTC, 50 ep) still leads on several datasets—but reaches a competitive band *without any encoder fine-tuning*, which is precisely what the five-alert zero-day setting requires. Future work includes principled ablations, deployment-grade robustness evaluation, and on-site SOC integration studies.

## Figures and Tables

**Figure 1:**
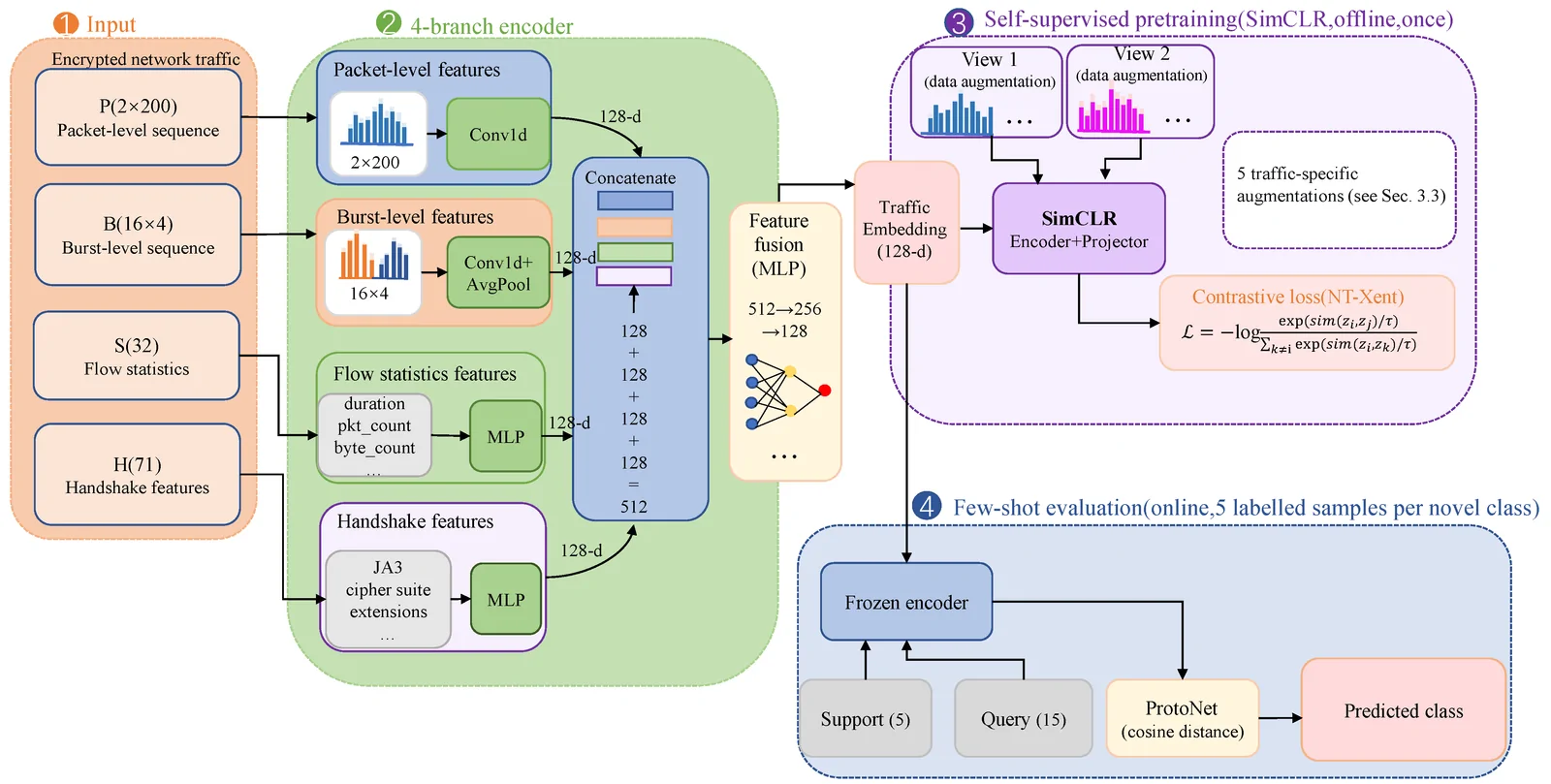
Overall architecture of TP-Net. Each flow is first decomposed into four feature views (packet, burst, flow statistics, TLS handshake). A four-branch 1D-CNN encoder maps these views to a 128-D embedding. The encoder is pretrained once, offline, under SimCLR with five traffic-specific augmentations, and it is then frozen; at deployment, a one-line logistic-regression head is fitted on *K* labeled support samples per novel class.

**Figure 2:**
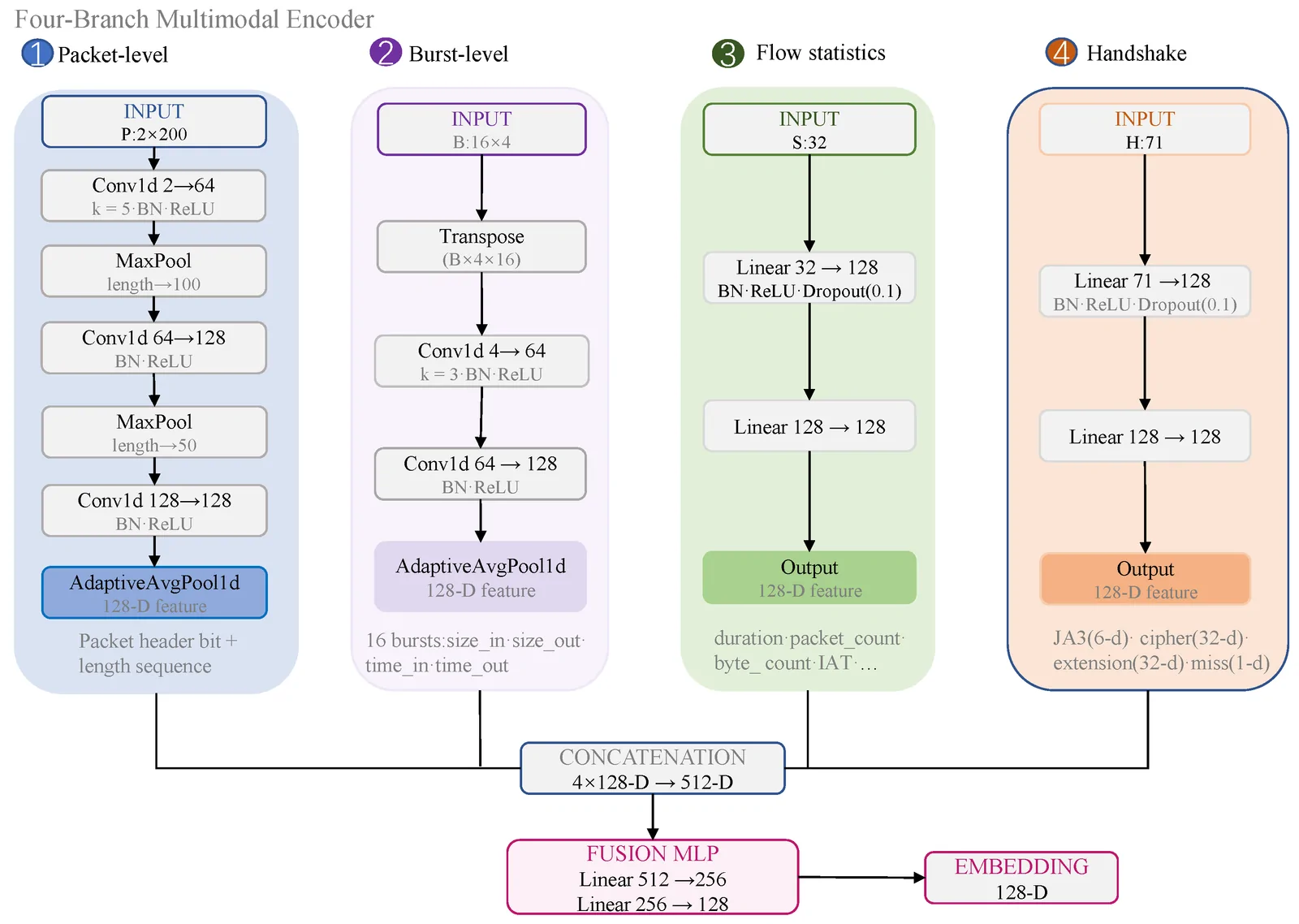
Detailed structure of the four-branch encoder.

**Figure 3:**
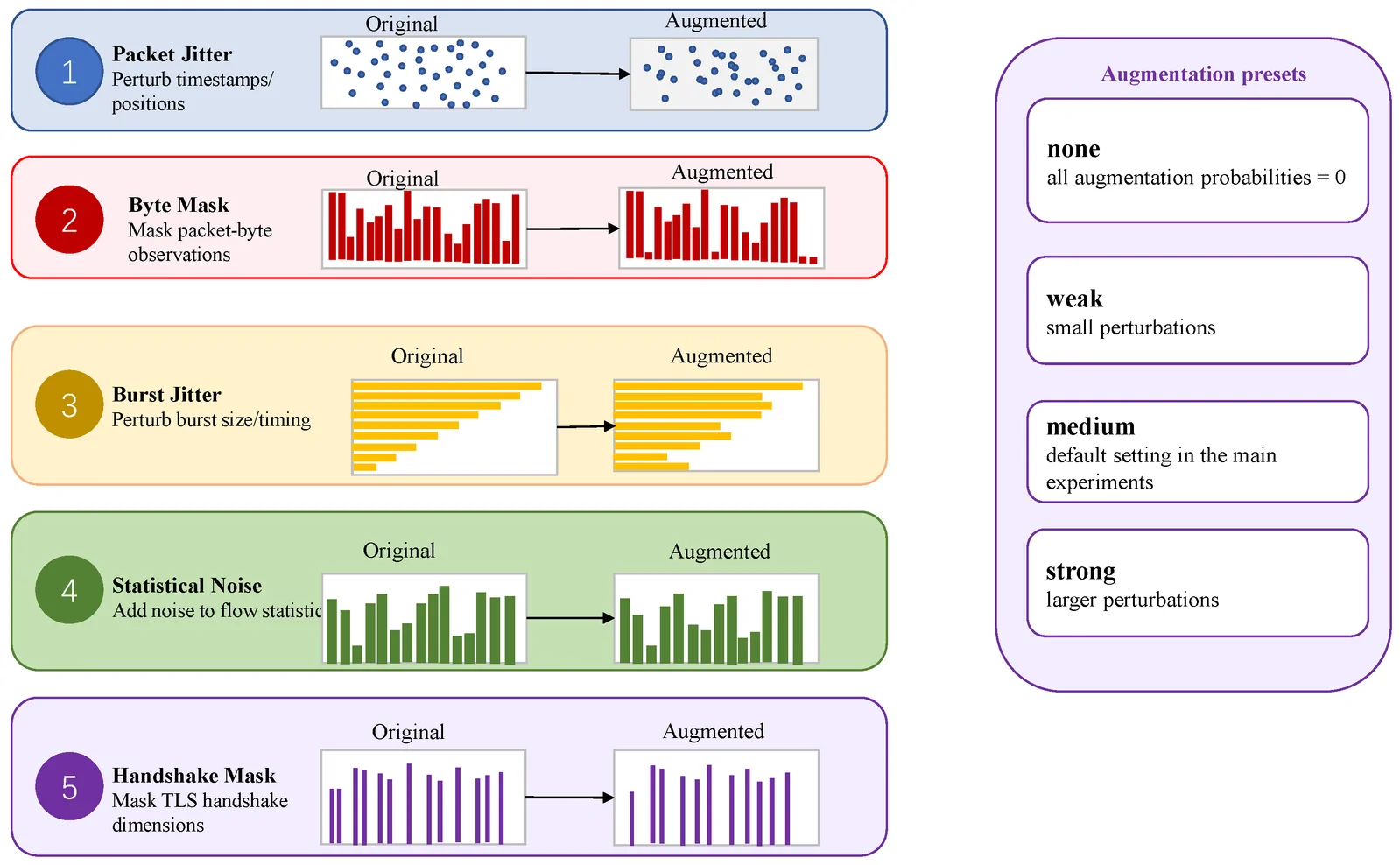
Schematic of the five traffic-specific augmentations (with the four intensity presets none/weak/medium/strong).

**Figure 4:**
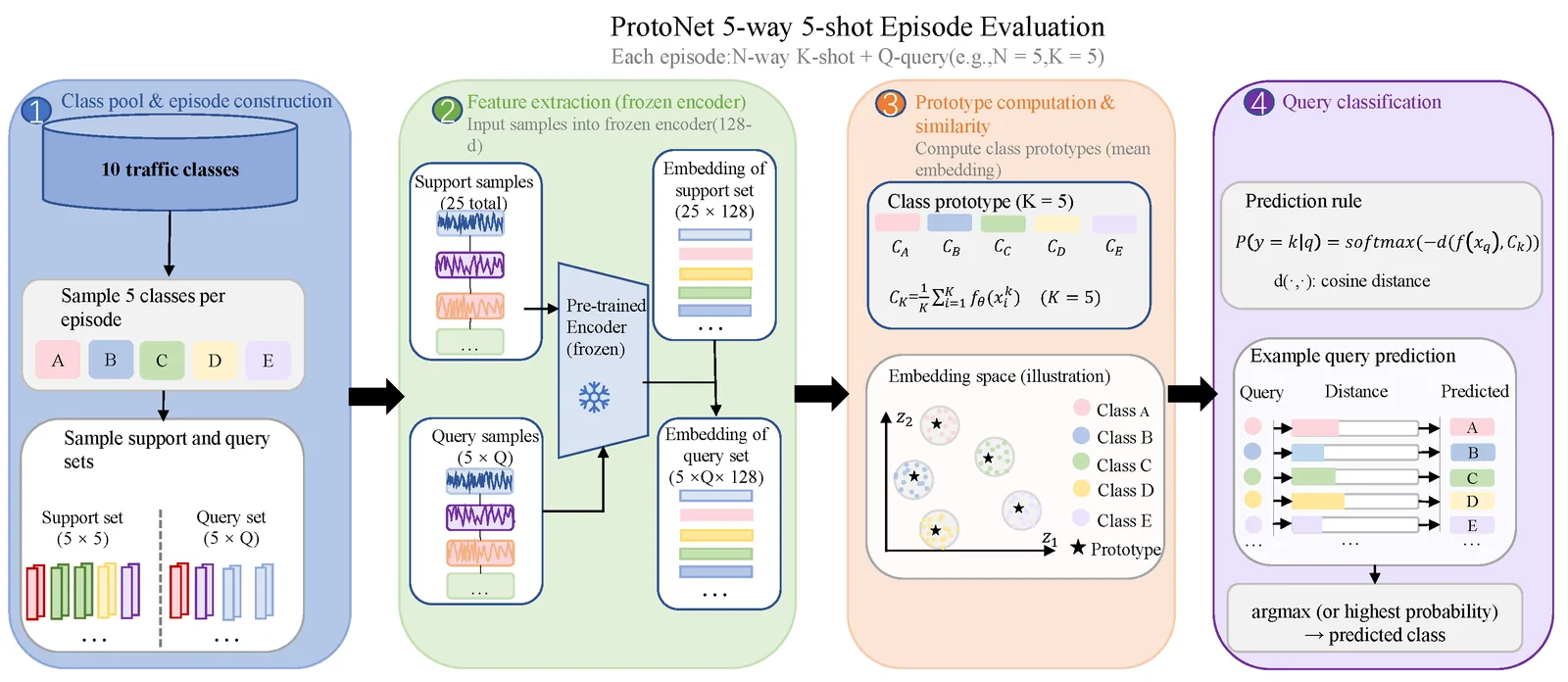
Flow of ProtoNet 5-way 5-shot episode evaluation.

**Figure 5:**
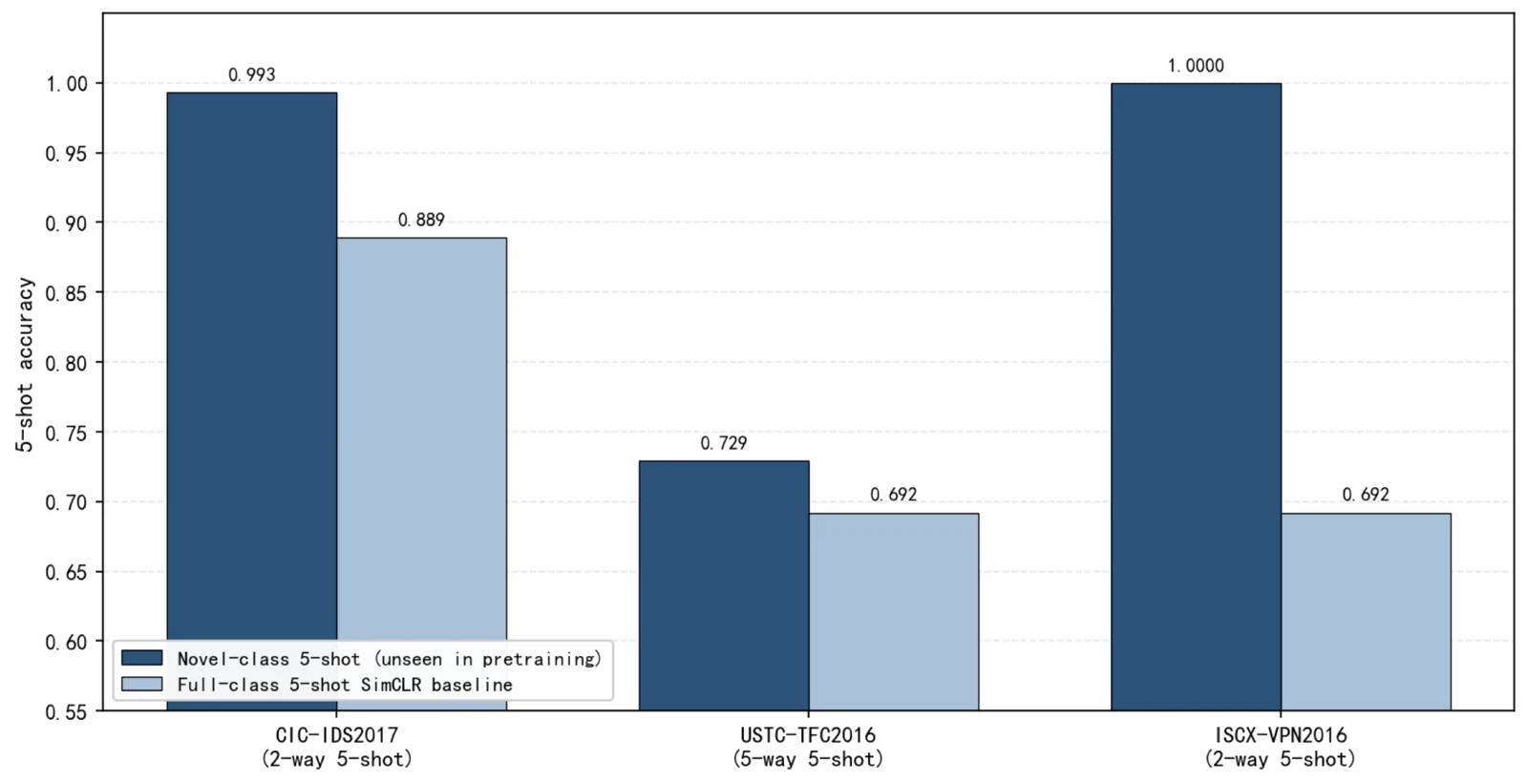
Novel-class 5-shot vs. all-class 5-shot SimCLR (dark-blue bars: novel classes unseen during pretraining, evaluated with 5 supports per class; light-blue bars: same-protocol all-class baseline; fully supervised LinearProbe is under a different protocol and is therefore excluded from this figure).

**Figure 6:**
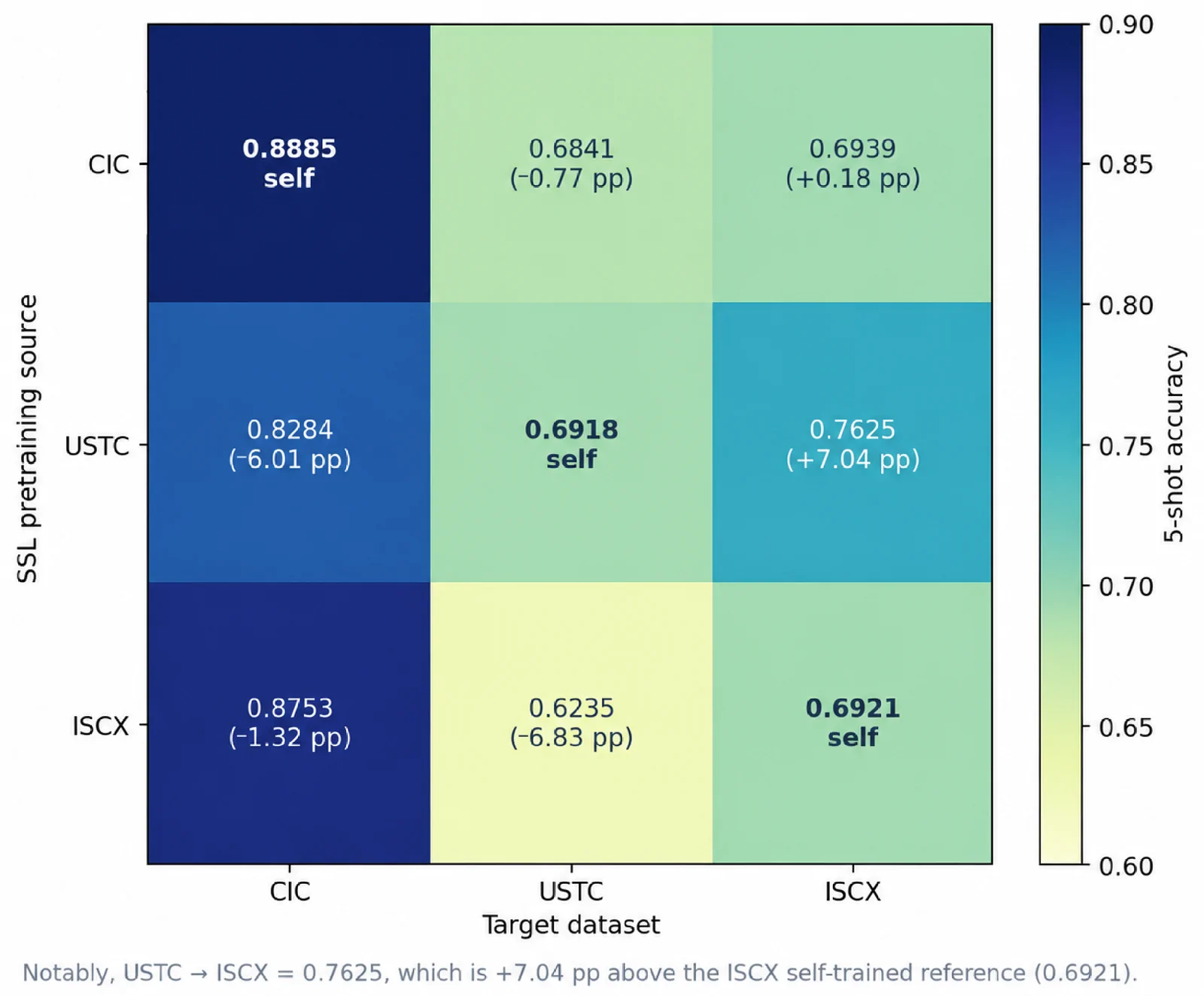
Cross-dataset transfer (full 3×2 matrix).

**Table 1:** Five-dimensional comparison of existing work and TP-Net.

Dimension	Existing Work	TP-Net (This Paper)
Learning paradigm	Fully supervised training	SSL pretraining + few-shot evaluation
Novel attack adaptation	Full retraining (hours–days)	5-shot labeling, hour-level deployment
Representation independence	Encoder tightly coupled to head	Frozen encoder, plug-and-play
Evaluation protocol	Fixed train/val/test split	*N*-way *K*-shot episode protocol
Data augmentation	CV-style flipping/cropping	Five traffic-specific, physically meaningful augmentations

**Table 2:** Datasets.

Dataset	Classes	Samples	Task	Encryption Coverage
CIC-IDS2017 [36]	10	1,084,344	Plaintext threats (HTTPS subsets)	Partial HTTPS
USTC-TFC2016 [37]	20	488,000	Semi-encrypted (incl. BitTorrent)	Mostly app-layer encryption
ISCX-VPN2016 [38]	2	333,123	VPN binary classification	All VPN tunnels

**Table 3:** Protocol correspondence across every model family used in the paper. All rows share a frozen encoder and an episodic *N*-way *K*-shot evaluation; the columns isolate what differs.

Model Family	Pretraining/Training	Encoder Status	Evaluation Head
TP-Net (SSL)	SimCLR, 50 ep (ISCX 30), 20k subset	frozen	ProtoNet/FT-Head LogReg
Random 1D-CNN	none	frozen (random init)	ProtoNet/FT-Head LogReg
Supervised baselines (short)	3 supervised ep, 20k subset	frozen	FT-Head LogReg
Supervised full budget (YaTC, TFE-GNN)	50 supervised ep, 20k subset	frozen	FT-Head LogReg
Novel-class TP-Net	SimCLR, 50 ep, base classes only	frozen	ProtoNet *N*-way 5-shot
Encrypted-only TP-Net	SimCLR (NT-Xent), 30 ep, DoHBrw-2020	frozen	ProtoNet 2-way 5-shot

**Table 4:** Main 5-shot results: SSL vs. random initialization (95% CI seed-level, n=5 seeds × 600 episodes). Bold indicates the best result.

Dataset	Task	Random	SimCLR + ProtoNet	SimCLR + LinearProbe	$\Delta_{\text{Proto-Rand}}$ (pp)
CIC-IDS2017	5-way 5-shot	0.7571 ± 0.006	**0.8885 ± 0.003**	**0.9972 ± 0.001**	+13.14
USTC-TFC2016	5-way 5-shot	0.6142 ± 0.002	**0.6918 ± 0.003**	0.5951 ± 0.008	+7.76
ISCX-VPN2016	2-way 5-shot	0.6510 ± 0.004	**0.6921 ± 0.003**	0.8637 ± 0.006	+4.11

Note: $\Delta_{\text{Proto-Rand}}$ = (SimCLR + ProtoNet) − Random in percentage points. CI is seed-level (n=5); the statistical unit is the seed—each of n=5 seeds evaluates 600 episodes and contributes one mean accuracy, so the CI and paired *t*-test are computed over n=5 seed-level observations; the many episodes are not independent, and treating them as such would inflate significance; all paired *t*-tests of SSL vs. Random yield p<0.001. The ProtoNet-vs.-MatchingNet gaps (+1.25/+7.15/+8.24 pp) and the SimCLR-vs.-MoCo gap on CIC (+1.35 pp) are tabulated in Table A3.

**Table 5:** Supervised baselines under FT-Head as an upper-bound reference for the same per-episode protocol. The SSL encoder’s main results appear in Table 4 (SimCLR + ProtoNet vs. Random, the canonical SSL gain) and Section 4.3 (novel-class generalization, the deployment scenario where SSL is uniquely valuable); Table 5 anchors where supervised pretraining with a per-episode linear head can reach under a comparable resource budget. Bold indicates the best result.

Method	Paradigm	CIC-IDS2017		USTC-TFC2016		ISCX-VPN2016
		5w5s	Δ vs. TP-Net FT-Head	5w5s	Δ vs. TP-Net FT-Head	2w5s
ET-BERT [6]	MLM + supervised	0.7961 ± 0.004	−12.52	0.4858 ± 0.004	−32.15	0.5017 ± 0.001
FS-Net [4]	Recon + supervised	0.5692 ± 0.006	−35.21	0.2001 ± 0.000	−60.72	0.6506 ± 0.005
YaTC [5]	MAE + Transformer	**0.9482**± 0.002	+2.69	**0.8397**± 0.004	+3.24	0.8391 ± 0.003
1D-CNN [41]	1D-CNN + supervised	0.9233 ± 0.003	+0.20	0.3305 ± 0.002	−47.68	0.6414 ± 0.005
NetMamba [10]	Mamba + supervised	0.8903 ± 0.002	−3.10	0.3439 ± 0.006	−46.34	0.7560 ± 0.004
TFE-GNN [7]	GNN + supervised	0.8337 ± 0.002	−8.76	0.8106 ± 0.004	+0.33	0.8240 ± 0.004
Random (no pretrain) + LogReg	—	0.7571 ± 0.006	−16.42	0.6142 ± 0.002	−19.31	0.6510 ± 0.004
**TP-Net (SimCLR ProtoNet)**	**SSL + ProtoNet**	**0.8885**± 0.003	−3.28	**0.6918**± 0.003	−11.55	0.6921 ± 0.003
**TP-Net (SimCLR FT-Head)**	**SSL + LogReg**	**0.9213**± 0.002	**—**	**0.8073**± 0.002	**—**	**0.7880**± 0.004
Full-budget sanity check(50 supervised epochs, two strongest baselines)	—	—	—	—	—	—
YaTC [5] (50 ep)	MAE + Transformer	**0.9897**± 0.001	+6.84	**0.8443**± 0.003	+3.70	**0.8855**± 0.005
TFE-GNN [7] (50 ep)	GNN + supervised	**0.9770**± 0.000	+5.57	**0.7994**± 0.003	−0.79	**0.7660**± 0.004
TP-Net (SimCLR FT-Head, single-stage, no fine-tune)	SSL + LogReg	0.9213 ± 0.002	—	0.8073 ± 0.002	—	0.7880 ± 0.004

Note: All methods use identical data, training budget (the number of supervised epochs on the same 20k stratified subset; 3 ep = resource-limited snapshot, 50 ep = full budget), frozen encoder, and per-episode sklearn LogReg ($C=10^{3}$) head (5 seeds × 600 episodes). Δ (pp) = baseline 5-shot accuracy − TP-Net SSL FT-Head 5-shot accuracy; positive values indicate the supervised baseline outperforms TP-Net on that column. Bottom block: 50-epoch sanity check for YaTC/TFE-GNN. Chance: 0.20 (5-way)/0.50 (two-way). Per-seed values are released in the public repository; see Section 4.2 for interpretation.

**Table 6:** Encrypted-only DoHBrw-2020 validation (2-way 5-shot, 5 seeds × 600 episodes, balanced *N* per episode). Bold indicates the best result.

Encoder	2-Way Chance	Mean Acc	Δ vs. Random (pp)	Notes
Random (no pretrain)	0.5000	0.5514 ± 0.012	—	Same arch., no SSL
TP-Net (SimCLR ProtoNet)	0.5000	**0.8380** ± 0.006	+28.66	NT-Xent, 30 ep
Higher-shot(1-way 10-shot, sanity)	—	0.8792 ± 0.004	—	Label-volume trend

**Table 7:** Three-dataset augmentation ablation (4 presets × 3 datasets, 12 data points, all 5-seed × 600 episodes). Bold indicates the best result.

Dataset	Task	None	Weak	Medium	Strong	BEST
ISCX-VPN2016	2-way 5-shot	**0.6950 ± 0.005**	0.5779 ± 0.005	0.6290 ± 0.005	0.6823 ± 0.004	**none**
CIC-IDS2017	5-way 5-shot	0.8425 ± 0.004	0.8793 ± 0.003	**0.8826 ± 0.003**	0.8806 ± 0.004	**medium**
USTC-TFC2016	5-way 5-shot	0.6538 ± 0.002	**0.6780 ± 0.002**	0.6703 ± 0.003	0.6642 ± 0.004	**weak**

**Table 8:** SSL vs. supervised, and ProtoNet vs. fine-tune head (5 seeds × 600 episodes; SSL SimCLR encoder frozen; FT-Head uses sklearn LogReg $C=10^{3}$). (**a**) Known classes. (**b**) Novel classes (SSL vs. Sup). Bold indicates the best result.

**(a)**					
**Dataset**	**N-Way K-Shot**	**Random**	**Sup**	**SSL Proto**	**SSL FT-Head**
ISCX	2w5s	0.6348	**0.8879**	0.6921	0.7880
CIC	5w5s	0.7140	**0.9353**	0.8707	0.9213
USTC	5w5s	0.6128	0.6994	0.6895	**0.8073**
**(b)**					
**Dataset**	**Scenario**		**Sup**	**SSL Proto**	
ISCX	novel 1/1, 1w5s		1.0000	1.0000	
CIC	novel 7/3, 2w5s		0.8118	**0.8463**	
USTC	novel 14/6, 5w5s		**0.6767**	0.6368	

Note: On known classes, Sup−SSL ProtoNet gaps are +19.58/+6.46/+0.99 pp (ISCX/CIC/USTC); FT-Head − ProtoNet gains are +9.59/+5.06/+11.78 pp. On novel classes, SSL > Sup on CIC (+3.45 pp) and Sup > SSL on USTC (+3.99 pp); ISCX is trivial (N=1). The novel-class panel uses a second partition distinct from the main split: CIC novel = BENIGN/DoS Hulk/PortScan; USTC novel = Geodo/SMB/Miuref/Nsis-ay/Skype/Htbot.

**Figure A1:**
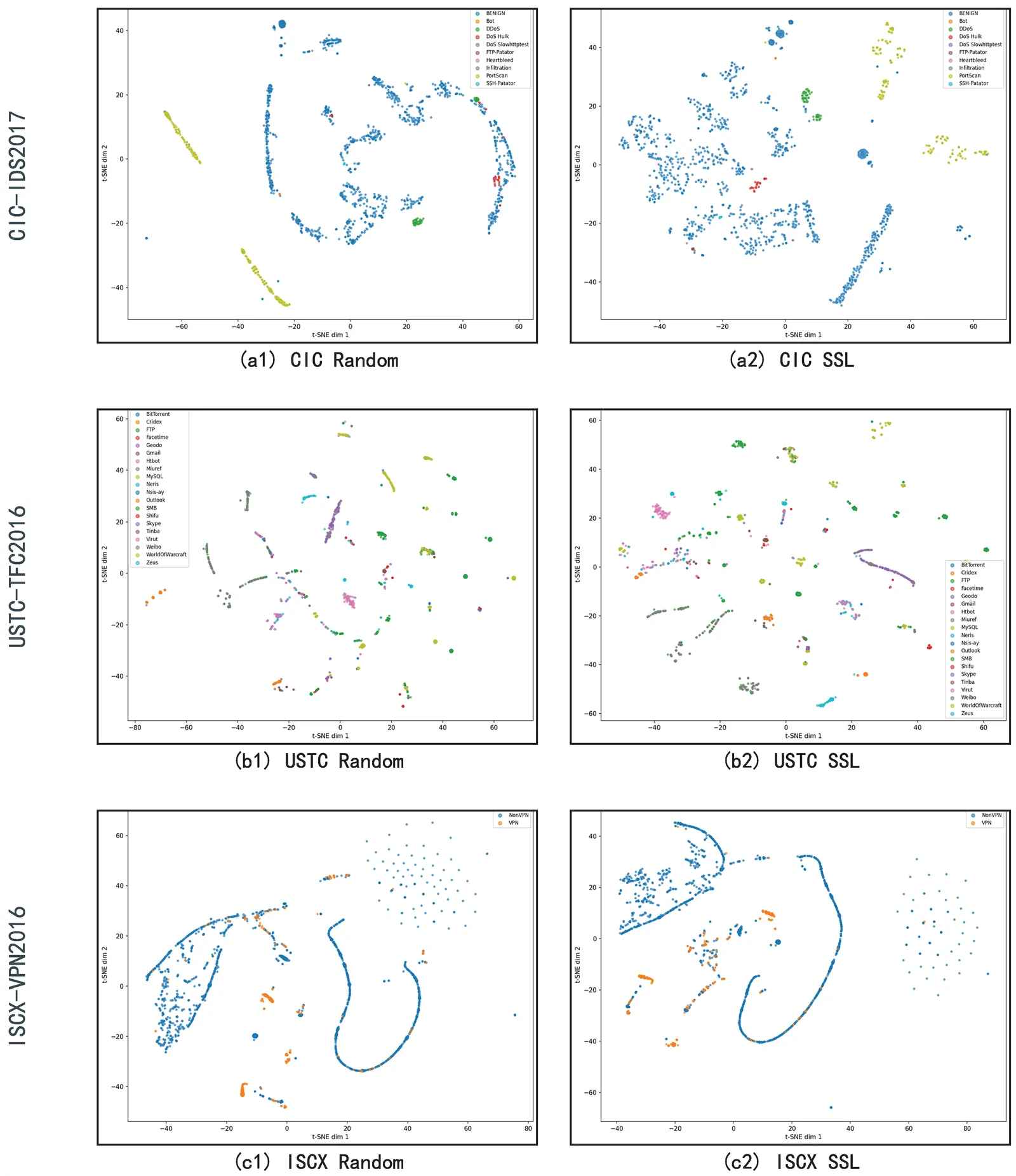
t-SNE projections (3 datasets × Random/SSL; subcaptions (**a1**–**c2**) at the same size as the figure caption).

**Figure A2:**
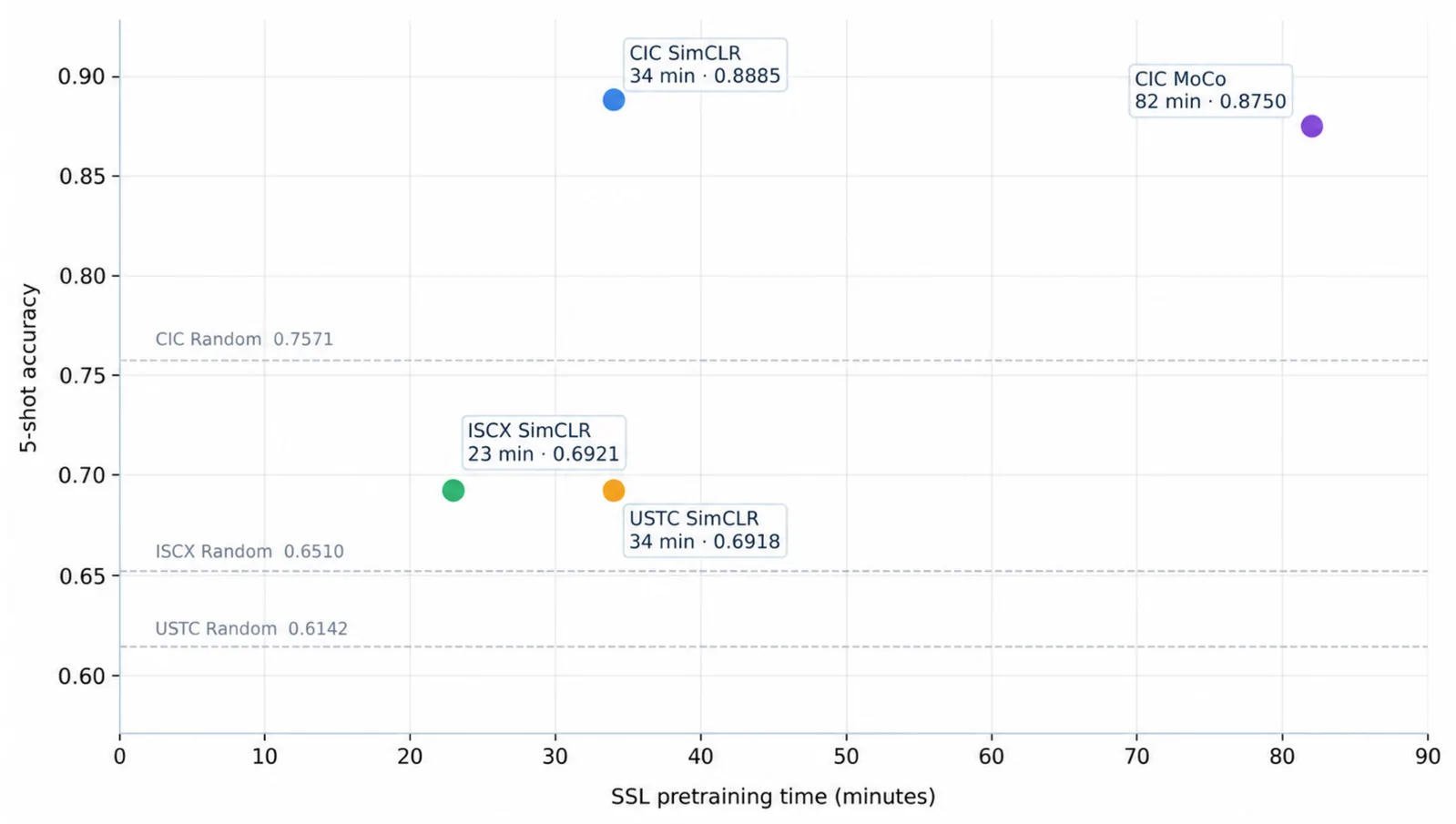
SSL pretraining cost–benefit scatter.

## Data Availability

The three public traffic datasets used in this work are openly available: CIC-IDS2017 (https://www.unb.ca/cic/datasets/ids-2017.html) (accessed on 2 October 2026), USTC-TFC2016 (https://github.com/yungshenglu/USTC-TFC2016) (accessed on 2 October 2026), ISCX-VPN2016 (https://www.unb.ca/cic/datasets/vpn.html) (accessed on 2 October 2026), and DoHBrw-2020 from the Stratosphere IPS Lab (https://stratosphereips.org/category/dataset.html) (accessed on 2 October 2026). All preprocessing scripts, pretrained SimCLR checkpoints (per dataset and SSL algorithm), and per-seed episode-level results are released at https://github.com/YUN625-art/TP-Net under an MIT license; the link will be replaced with the final archival URL upon acceptance.

## Abbreviations

The following abbreviations are used in this manuscript:

AUROC	Area Under the Receiver Operating Characteristic Curve
BYOL	Bootstrap Your Own Latent
CDTF	Contrastive Dual-Task Framework
CI	Confidence Interval
CKA	Centered Kernel Alignment
CNN	Convolutional Neural Network
DoH	DNS-over-HTTPS
DoHBrw	DNS-over-HTTPS Browsing dataset
DoS	Denial of Service
DPI	Deep Packet Inspection
FSL	Few-Shot Learning
GNN	Graph Neural Network
IDS	Intrusion Detection System
IoT	Internet of Things
JA3	TLS Client Fingerprint
MAE	Masked Autoencoder
MAML	Model-Agnostic Meta-Learning
MEC	Multi-access Edge Computing
MLM	Masked Language Modeling
MoCo	Momentum Contrast
NT-Xent	Normalized Temperature-scaled Cross-Entropy
ProtoNet	Prototypical Network
SimCLR	Simple Contrastive Learning of Visual Representations
SSL	Self-Supervised Learning
TFE-GNN	Traffic Feature Extraction Graph Neural Network
TLS	Transport Layer Security
TP-Net	Traffic Pretraining Network
WSN	Wireless Sensor Network
YaTC	Yet Another Traffic Classifier

## Appendix A. Supplementary Experiment Tables and Visualizations

The appendix tables and figures cited in the main text are collected below.

**Table A1:** Full *K*-shot scan (3seeds×200episodes; **note: this appendix table uses a smaller protocol than Table 4 (5 seeds × 600 episodes)**; the K=5 row here is consistent with Table A2 but differs from Table 4 by sample-size variance, cited in Section 4.2).

Dataset	K-Shot	1	3	5	10
CIC-IDS2017	SSL	0.8521	0.8624	0.8700	0.8752
	Random	0.7728	0.7366	0.7115	0.6799
	Δ (pp)	+7.93	+12.58	+15.85	+19.53
USTC-TFC2016	SSL	0.6621	0.7258	0.7391	0.7525
	Random	0.6995	0.7376	0.7465	0.7498
	Δ (pp)	−3.74	−1.18	−0.74	+0.27
ISCX-VPN2016	SSL	0.6786	0.6407	0.6462	0.6443
	Random	0.6328	0.6266	0.6419	0.6484
	Δ (pp)	+4.58	+1.41	+0.43	−0.41

**Table A2:** SSL algorithm diversity (ProtoNet *N*-way *K*-shot, 3seeds×200episodes, ISCX is 2-way; **note: this appendix table uses a smaller 3 × 200 protocol than Table 4; absolute numbers therefore differ slightly from Table 4 but the ranking is preserved**; ranking is discussed in Section 3.4). Bold indicates the best result.

SSL Algorithm	Paradigm	CIC-IDS2017	USTC-TFC2016	ISCX-VPN2016	3-Dataset Mean
**SimCLR**	Contrastive (in-batch negatives)	**0.8700 ± 0.004**	**0.7391 ± 0.008**	**0.7026 ± 0.008**	**0.7706**
MoCo	Contrastive (momentum queue)	0.8537 ± 0.002	0.6606 ± 0.006	0.6249 ± 0.012	0.7131
BYOL	Bootstrapping (EMA target)	0.8676 ± 0.001	0.6802 ± 0.004	0.6104 ± 0.010	0.7194
SimSiam	Bootstrapping (stop-gradient)	0.8465 ± 0.003	0.6592 ± 0.006	0.5573 ± 0.013	0.6877
Embedding-MAE †	Reconstructive (embed. mask)	0.7886 ± 0.003	0.6121 ± 0.005	0.5806 ± 0.011	0.6604

Note: † Embedding-MAE (simplified version in this paper) ≠ the MAE of He et al. [19]; see Section 3.4.

**Table A3:** Extended SimCLR heads comparison (MoCo/MatchingNet columns removed from Table 4 for compactness; full data restored here, 5 seeds × 600 episodes).

Dataset	Task	SimCLR + MoCo	SimCLR + MatchingNet	$\Delta_{\text{Proto-Match}}$ (pp)
CIC-IDS2017	5-way 5-shot	0.8750 ± 0.003	0.8760 ± 0.003	+1.25
USTC-TFC2016	5-way 5-shot	0.5880 ± 0.005	0.6203 ± 0.005	+7.15
ISCX-VPN2016	2-way 5-shot	0.6223 ± 0.003	0.6097 ± 0.003	+8.24

Note: $\Delta_{\text{Proto-Match}}$ = (SimCLR + ProtoNet) − (SimCLR + MatchingNet), i.e., the metric-learning gain over cosine attention. SimCLR > MoCo by +1.35 pp on CIC (0.8885 vs. 0.8750); MoCo is worse than Random on USTC/ISCX.

**Table A4:** USTC per-class 5-shot accuracy (SSL vs. Random, 5 seeds × 600 episodes; top 5 + bottom 5, 10 classes, cited in Section 4.4). Bold indicates the best result.

Application Class	SSL	Random	Δ (pp)
— SSL top 5 (SSL strongest) —			
Outlook	0.9906	0.9920	−0.14
WorldOfWarcraft	0.9822	0.9999	−1.77
Tinba	0.9280	0.3932	**+53.48**
Skype	0.9021	0.9142	−1.21
Facetime	0.8822	0.8891	−0.69
*— SSL bottom 5 (SSL weakest) —*			
BitTorrent	0.5196	0.5845	−6.49
Neris	0.5153	0.3855	**+12.98**
Gmail	0.5120	0.5164	−0.44
Miuref	0.5024	0.3135	**+18.89**
Nsis-ay	0.5007	0.2413	**+25.94**

**Table A5:** Open-set FSL evaluation (CIC/USTC, 3 seeds × 200 ep, cited in Section 5.2). Bold indicates the best result.

Dataset	Scenario A	Scenario B	Scenario C	Scenario B	Scenario C
	Closed-Set Acc	AUROC	AUROC	Conf Gap	Conf Gap
CIC-IDS2017	0.8261	**0.9127 ± 0.004**	0.8285 ± 0.002	**0.51**	0.29
USTC-TFC2016	0.8736	0.8022 ± 0.003	0.7202 ± 0.008	0.07	0.07
